# Indents

**Published:** 1922-05

**Authors:** 


### INDENTS

**☞Brief Articles of Technical or Scientific Value will receive notice and comment. Contributions invited.**

---

FIGURES NEVER LIE—BUT FIGURERS DO. Those practitioners of the healing art who maintain that all pathologic conditions, from cancer to chilblains and from soft corns to hardening of the liver, are due to subluxated vertebrae impinging on spinal nerves are republishing their annual batch of ''statistics'' on the chiropractic treatment of influenza. The standard advertisement runs, in part, as follows:

The Following Statistics of the 1918 ''Flu'' Epidemic are Respectfully Submitted:

One of Every 16 Patients Died Under Medical Treatments.
One of Every 127 Patients Died Under Osteopathic Treatments.
One of Every 513 Patients Died Under Christian Science Treatments.
One of Every 886 Patients Died Under Chiropractic Adjustments.

These figures, of course, are evolved from the inner consciousness of those gentlemen that furnish verbal ammunition for chiropractic advertising campaigns. But, even assuming them to be correct, just what do they prove? They prove that many more people die when under the care of a physician than die when under the care of an osteopath, a Christian science practitioner or a chiropractor. The medical profession is perfectly willing to admit this; it is equally willing to admit that the vast majority of those who die, die in bed. Neither of these somewhat self-evident propositions, however, argues that scientific medicine is more dangerous than chiropractic, ''Christian science'' or osteopathy, or that a bed is a dangerous place. They do prove that most people who are sick enough to be in danger of death are usually in bed and under the care of a physician. Any one who is familiar with the facts may admit that comparatively few people die while directly under "chiropractic adjustment" or any other of the fad "treatments." There are two outstanding reasons for this. The first is that the man who relies, for example, on chiropractic for the relief of some passing indisposition precipitately deserts this cult when he realizes that he is dangerously ill. Then he calls in a physician; should he die, he dies under "orthodox medical treatment." The second reason is that, should a patient die under "chiropractic adjustment," the law would require an inquest, as in very few states in the Union are these gentry permitted to sign death certificates. It is notorious that when the "patient" of a chiropractor becomes dangerously ill, the chiropractor urges the family to call in a physician!—*Journal A. M. A.*

---

Suggestions for the Cleaning Up of Gold Nuggets. For cleaning up gold nuggets the following method has been found to be satisfactory. A depression should be made in a charcoal block so that the walls of the depression stand above the nugget. Slightly heat the gold with the flame and then sprinkle on a mixture of equal parts, by volume, of powdered borax and powdered charcoal. Use this flux generously and apply it as heating is continued. When the nugget is melted apply borax and charcoal mixture so that the gold is covered, allow to solidify and then with forceps turn the nugget over and repeat the process. When nugget has cooled, break or knock off the borax and then place in a warm or hot thirty per cent. solution of sulfuric acid. Upon removing the gold from the acid solution it should be dipped in a solution of bicarbonate of sodium in order to neutralize the acid.

In case the nugget has been contaminated with a somewhat refractory metallic oxide, the borax-charcoal method will not be sufficient. In such a case the metal when molten should be generously sprinkled with a mixture of one-quarter powdered ammonium chloride and three-quarters powdered borax. Follow this treatment with the borax-charcoal method.

The flame alone can be employed with success. To any flame there is a reducing and an oxidizing atmosphere. The oxidizing part is found near the end of the flame and in this part all material which burns up or oxidizes rapidly combines with oxygen. Further back from the tip will be found the reducing atmosphere. In this part metals are prevented from oxidizing and any oxidized metal is reduced, due to presence of free hydrogen in the flame.

To find these parts in the flame take a piece of gold which contains copper, for instance 22k. dark gold, and heat to redness with the front part of the flame. Allow gold to cool and heat again, this time playing the flame on the gold at a point nearer the burner tip. By working to and fro a position will be noticed at which the surface of the gold is brightened and therefore free from oxides. This is the reducing part of the flame. A moderate flame should be used. The hydrogen in the flame reduces the metallic oxide to the metal. The use of borax-charcoal is suggested as previously described.

Another method, found to give very good results, is by the use of a crucible. The following things are necessary: a hard sand crucible and cover, a tripod, a carbon stick and a blast lamp or any good large, hot flame. Place the crucible in the tripod in such a manner that it is suspended from a point near the top. Heat the crucible slowly, using great care, as too sudden heating will cause it to crack. When the crucible is red, look into it and if no crack is visible place the gold nuggets or scrap (any gold except solder or clasp metal), in the crucible and cover with a layer of charcoal. Heat until gold is molten, stir with a carbon stick and then blow off nearly all the charcoal. Add a quantity of borax, enough to cover the gold well, heat again, and after molten for a few moments, pour out into an oiled mold or into a depression in a charcoal block.

The use of oxidizing agents, such as sodium nitrate or potassium chlorate, is not recommended. It serves only to assist in removing foreign material. It is harmful in that it induces oxidation of gold alloys.

Gold alloys should not be placed in acid while they are red hot. The metal in this state is expanded and the tiny pores will occlude acid when rapidly cooled in it. It is better to place cooled gold in hot acid.—L. R. Hess, in *Pacific Gazette*.

---

THE CHOICE OF ANESTHETICS IN EXTRACTION. The use of local anesthetics for operations on infected tissues where the anesthetic, to a greater or lesser extent, interferes with the circulation to the part to be operated on, either by infiltration or nerve-blocking, produces the same condition artificially as exists in bruised tissue, namely, interference with the circulation and oxygen tension, and thereby lowers the resistance of that tissue to invading bacteria.

A bloodless field for the removal of teeth has been the aim of many operators and the condition very much desired by men who are using local anesthetics. While a bloodless field is a great help to an operator, it in the other hand is a great detriment to the patient, *first*, because it lowers the resistance of that tissue to invading pathogenic microorganisms, and, *second*, because it interferes with the normal coagulation time of the blood, which is nature's protection for any wound.

Professor Martin H. Fischer, who has so brilliantly investigated the relation of deoxygenation and edema to all conditions of systemic infection, writes me as follows: "Where local anesthetics are used the operator should cut down the amount of adrenalin used to the minimum, and also the total amount of local anesthetic, try to inject the anesthetic where it will hit a nerve trunk. In other words, *produce the maximum of anesthesia with a minimum of local ischemia.* Of course, the nitrous oxid-oxygen anesthesia is the anesthetic of choice. The trouble is that dental operators either do not know how to use it properly and do not get enough time, or else are bad operators from the start and so do not make a good job of an infected area in the briefer time of unconsciousness and insensibility to pain allowed them with nitrous oxid-oxygen. I, too, have seen bad systemic infections follow what I call these bungled mouth operations. They are very common, too, when operations are done badly on the tonsils, and here it is the same story. Unless local anesthetics are most carefully used and trauma is reduced to a minimum, there is much subsequent swelling of the injured part and great liability to infection.

I do not know exactly, of course, why the infection occurs more easily in the injured tissues, but I am inclined personally to the view that the lack of oxygen in the swollen tissues, aided, of course, by the good culture ground of blood and bruised tissues, tells the whole story. All we have to do is to shrink their swollen tissues and let oxygen get at the bacteria and they die. What is the best, for instance, for the treatment of 'rheumatism' is air, caffein and alkali, and wet dressing with magnesium sulphate, and what does all this mean, excepting systematic alkalinization, improvement in oxygen supply to the affected parts, and natural death of the infecting organisms?"

Teeth which have diseased root-ends, such as a granuloma, are more or less walled-off by nature from the surrounding tissue, and it is desirable that when these teeth are removed, as little auto-inoculation as possible should be given to the surrounding healthy tissue and the healthy tissue should not be placed in a position of lowered resistance.

Regardless of what surgical technique is employed for the removal of these teeth and their granulomata, is it not reasonable to suppose that if the surrounding tissue is left in its highest state of immunity the so-called reaction or exacerbation will be lessened?

In those fatal cases which have been reported because of having too many teeth removed at one sitting, in only one case has any mention been made of the anesthetic used, and in that case the anesthetic was ether, and it was admitted that the patient had nephritis to begin with, which was, no doubt, aggravated by the ether anesthesia and not the removal of the infected teeth.

### CONCLUSIONS

(1) For the well-being of the patient anesthetics are not properly considered as to their physiological and pathological action.

(2) In the removal of diseased teeth it is desirable that nine of the activity of the body defenses be limited or destroyed by the use of any drugs or anesthetics.

(3) The leukocytes are the body's first line of defense against invading micro-organisms.

(4) Any agent which limits the action of the leukocytes, either by destroying them or preventing their passage to the invaded part by initial ischemia and secondary edema, tends to lower the resistance of that tissue and the individual to the invading micro-organisms.

(5) Any tissue which has had its normal circulation and oxygen tension interfered with has lost its normal degree of immunity.

(6) Local anesthetics initially interfere with the circulation and oxygen tension of the parts that they anesthetize, and, secondarily, cause edema, thereby increasing the susceptibility of the part to infection and auto-inoculation.

(7) General anesthetics, other than nitrous oxid and oxygen, lower the patient's resistance to infection.

(8) Fatal cases from the removal of infected teeth have not been properly reported.—B. H. Harms, *Cosmos,* April, 1922.

---

THE TEETH AND TUBERCULOSIS. A rigorous care of the mouth and teeth is necessary in the treatment of tuberculosis. In fact, the condition of the mouth is very impor-
from the viewpoints of diagnosis, prognosis and thera-
peutics. As a physician requires the assistance of a dentist in the treatment of certain systemic diseases, the
tant from the viewpoints of diagnosis, prognosis and thera-
dentist needs the assistance of the physician in the treatment of certain dental diseases. The closest co-operation should exist between the two professions.—H. Brady, D.M.D, *Cosmos,* p. 412, April, 1922.

---

FORMULA OF THE FLUID FLUX THAT DOES NOT PIT. Powdered Borax, 7 drachms; Powdered Boracic Acid —C. P., 7 drachms. Put all in a pint bottle and add: Distilled *cold* water, 6 ounces. Shake well until all is dissolved; then filter, pouring back the liquid, until perfectly clear. Put in a 6-ounce bottle and label: "*Fluid Flux.*" Besides this, take a 1-ounce "pomade" bottle for use in the laboratory.—*Oral Health.*

